# Research-Based Theater and “Stigmatized Trauma”: The Case of Suicide Bereavement

**DOI:** 10.3389/fpsyg.2020.01129

**Published:** 2020-06-16

**Authors:** Anneli Silvén Hagström

**Affiliations:** Department of Social Work, Stockholm University, Stockholm, Sweden

**Keywords:** bereavement, meaning-making, narrative, self-formation, stigma, suicide, theater

## Abstract

**Background:**

Existing research shows that family members who suffer the loss of a loved one through suicide often experience self-blame and shame, and that this limits their grieving process. It can also lock them into stigmatized positions and the notion that either *somebody* or a *dysfunctional family* is to blame for the suicide.

**Aim:**

This article investigates from a narrative perspective how a theater play might counteract the stigma that surrounds suicide bereavement by contributing destigmatizing understandings of suicide.

**Methods:**

A theater play was performed in a churchyard theater in Stockholm, Sweden, in 2019. Audience members were asked to write down their free reflections on a form distributed at the theater. In particular, they were asked to assess *whether* they found the play related to their own lives and, if so, *how*; and to describe *what* they had learned. Their written reflections [*N* = 41] were analyzed from a narrative methodological perspective to investigate their responses to the play. Three categories of audience member were identified from their responses: people with their own suicide bereavement experiences; people with similar but different experiences of stigmatized trauma; and people who did not report any experiences of suicide or stigmatized trauma.

**Results:**

The suicide-bereaved generally reported familiarity with the thematic performed, in particular the “why question,” the blame and shame responses and the silenced family communication. Most of these aspects were also shared by those affected by other types of stigmatized trauma. Respondents from all categories emphasized how they had learned that suicide is a desperate rather than a deliberated act, caused by overwhelming emotional pain or depression. Ultimately, suicide was perceived as an involuntary death caused by complex interacting factors linked to both inner vulnerabilities and stressful life events, for which no one was to blame.

**Conclusion:**

The results show that research-based theater isa time-limited and cost-effective method of introducing alternative meanings and identities to both individual mourners and the broader cultural context from which stigma originates, and how it can have destigmatizing effects on a stigmatized trauma such as suicide bereavement.

## Introduction

### Rationale

It has been established in research that stigmatizing notions of suicide constitute a particular circumstance that suicide-bereaved family members need to manage during their grief, in addition to the loss itself (Sudak et al., 2008; Jordan and McIntosh, 2011). People bereaved by a suicide in the family testify in several studies how they feel ashamed and tainted by the suicide, and that their self-perception is negatively affected as a result (e.g. Sterner Demi and Howell, 1991; Cvinar, 2005; Sveen and Walby, 2008; Feigelman et al., 2009; Peters et al., 2016; Silvén Hagström, 2016). This stigmatization process has been described as dual, since a socio-cultural pressure is exerted on the suicide-bereaved from both within and without (Dunn and Morrish-Vidners, 1988; Silvén Hagström, 2013). The mourners themselves perceive suicide as a morally deviant death and ask themselves *why* their loved one *chose* to die (Loy and Boelk, 2014; Silvén Hagström, 2019). They also commonly take on the blame and shame for failing to prevent the suicide – or even for causing it (Cvinar, 2005; Peters et al., 2016; Silvén Hagström, 2016). At the same time, suicide-bereaved family members describe in research the negative responses they encounter within their social networks after the suicide (Allen et al., 1994; Chapple et al., 2015; c.f., Sheehan et al., 2018). Social insecurity (Dyregrov, 2004) or “empathic failure” (Feigelman et al., 2009) linked to stigmatizing interpretations of suicide make it difficult to acknowledge the family members’ grief and offer them support. This is also known as “disenfranchised grief” (Neimeyer and Jordan, 2002). The suicide-bereaved commonly report how individuals in their social circle avoid communication about the deceased family member and the suicide, and how a tense atmosphere usually arises when such topics are initiated by the bereaved themselves. It has also been reported that people distance themselves to avoid confronting the circumstances of the suicide, and how negative responses can also be directed toward the bereaved family, such as anger, explicit accusations and outright rejection (Allen et al., 1994; Feigelman et al., 2009; Maple et al., 2010; Silvén Hagström, 2013; Peters et al., 2016). All these non-supportive responses reinforce an already stigmatized position in grief, and make it difficult for the bereaved family to *talk* about the suicide and process the loss.

The understanding of suicide as an anomalous behavior, where blame is to be attributed to an immoral individual or a dysfunctional family, is rooted in Western culture (Minois, 1999; Cvinar, 2005). In the Middle ages, suicide was conceived as a banned act; and as both a religious sin – since only God has the power to give and take away life – and a criminal offense. Suicide and suicide attempts were associated with severe penalties; for example, the deceased through suicide was not allowed to be buried in consecrated ground. Sanctions were also directed at grieving family members, who commonly had to pay an extra tax to the church and were socially excluded from the community (Cvinar, 2005). Such authoritarian practices no longer apply in contemporary Western societies, but recent research, as shown above, demonstrates how stigmatizing practices still exist. This indicates that prevailing understandings of suicide as a deviant and morally norm-breaking death caused by individual responsibility still operate in social interactions, and the question of *who* is to blame for suicide is still present in the Western cultural meaning-making of suicide (Silvén Hagström, 2016; c.f. Turchi et al., 2019).

Taken together, since suicide-bereaved family members suffer emotionally and commonly lack social contexts for support, they are particularly vulnerable to developing psychological ill-health themselves, such as complicated grief, depression and suicidal behavior – and even to suicide (Runeson and Asberg, 2003; Cerel et al., 2008; Feigelman et al., 2009). It is therefore vital to equate postvention efforts directed at relatives affected by suicide with suicide prevention (Andriessen, 2009; Jordan, 2017). This also highlights the urgency of finding new ways to understand and facilitate grieving processes among the suicide-bereaved to promote their health and wellbeing. This article assumes, based on research, that stigmatization hinders the grieving process after a suicide in the family. It aims to investigate from a narrative perspective how a theater play might counteract stigma in suicide bereavement by contributing destigmatizing understandings of suicide.

### A Social Constructionist and Narrative Perspective on Suicide Bereavement

When striving to counteract the adverse health effects of suicide on mourning family members, it is important to understand the psychosocial processes of suicide bereavement. The present article is grounded in a social constructionist and narrative theoretical perspective on loss, grief and trauma (Neimeyer and Sands, 2011; Neimeyer et al., 2014). This conceptualization is based on Parkes’ (1971) theory of how a traumatic experience fits into an individual’s existing “assumptive worlds” and Janoff-Bulman’s (1992) theory on “shattered assumptions.” According to these theories, traumatic events can shatter basic, taken-for-granted assumptions about the life world and the self, as it is no longer possible to identify with them. Such basic assumptions commonly suggest that the world is a benevolent, fairly safe and predictable place, and that the individual as a member of it is meaningful and morally worthy. Non-normative deaths – and suicide in particular – are by their nature more incomprehensible and thus represent a greater challenge, and usually provide a “shock effect” to the mourner’s existing world of meaning (Neimeyer and Sands, 2011; Sands et al., 2011 p. 249). The mourner’s ongoing autobiography is thus disrupted and contested by the suicide event, which causes a potential *crisis of meaning* (Neimeyer and Sands, 2011; c.f. “biographical disruption” and chronic illness Bury, 1982).

Thus, grieving is mainly understood as a narrative process that aims to construct new meanings and identities in the wake of profound loss. Just as with stigmatization, such meaning reconstruction is a process that occurs both *within* and *between* people. The social aspects of this process correspond with the need to search for significant personal meaning while at the same time attempting to validate this meaning in social interactions with others. Accordingly, grieving is described as: “a situated interpretive and communicative activity charged with establishing the meaning of the deceased’s life and death as well as the post-death status of the bereaved within the broader community concerned with the loss” (Neimeyer et al., 2014, p. 485). Meaning can, however, also be searched for and negotiated outside of such immediate social networks, and through the use of artistic forms of expression such as writing, painting or participating in theater (Silvén Hagström, 2014; Thompson and Neimeyer, 2014).

From this social constructionist and narrative theoretical viewpoint, it is assumed that the meaning which mourners find through such situated, interpretive and communicative activities is either necessarily congruent with the meanings that permeate the larger sociocultural context, or represents an active form of resistance against them (Currier et al., 2006). Since the theater play analyzed contributed destigmatizing understandings of suicide, it is suggested that it should be viewed as a counter-narrative to existing prejudicial perceptions of suicide that permeate society (Andrews, 2004; c.f. Silvén Hagström, 2014). As such, it could potentially affect both an individual audience member’s sense-making of suicide, and negative attitudes to suicide more generally.

### Suicide as a Case of “Stigmatized Trauma”

An inhibitory factor for narrative meaning reconstruction in suicide bereavement is stigmatization (Silvén Hagström, 2016). Goffman (1963) defined stigma as a process of identification and othering, whereby individuals who are associated with a stigmatized condition through social interaction are discredited and positioned with an undesirable social status. Nowadays, the terms *stigma through association* or *associative stigma* are used with reference to an extended stigma that denotes the negative attitudes and actions taken against the family members, friends or others linked to a socially stigmatized individual (Burk, 2007; van der Sanden et al., 2015). The term *family stigma* has also been used to explain the gossip, patronizing remarks, criticism, unsolicited advice or avoidance directed at family members of individuals who belong to a stigmatized group, such as those suffering from psychological problems (Larson and Corrigan, 2008; Moses, 2014). In the case of suicide bereavement, in addition to the reported suffering of public stigma that appears in responses in the mourners’ social lives, grieving family members also commonly exercise *self-stigmatization* (Dunn and Morrish-Vidners, 1988). This is a process by which the same sociocultural understandings of suicide are internalized by the bereaved and directed toward themselves as a form of self-criticism (Cvinar, 2005; Feigelman et al., 2009; Silvén Hagström, 2013; Silvén Hagström, 2016). Such dual stigmatizing processes have been discussed as a potential contributory factor to suicide (Schomerus et al., 2015). Hence, self-stigma can induce negative emotional reactions, social withdrawal and feelings of hopelessness, which in turn can lead to the individual experiencing reduced social belonging and difficulties talking about the psychological distress. In addition, both public stigma and self-stigma are associated with reduced willingness to seek professional help (Schomerus et al., 2015).

In this article, suicide is understood to result in the tentative concept of “stigmatized trauma”. This concept indicates that suicide is usually perceived as a traumatic loss that induces stigmatizing responses within the suicide-bereaved family members and their social circle. In line with the above, stigma is assumed to constitute a barrier to communication and help-seeking that can negatively affect the grieving process by complicating the construction of manageable meanings and moral identities in the wake of suicide. Other cases of stigmatized trauma are substance abuse, domestic violence and sexual assault, as well as many other life- and self-changing events, that for reasons of self-blame and shame are usually surrounded by silence.

### Theater as a Means of Combating Stigma

Theater has a long history of being considered a means of contributing to psychological, interpersonal and social change. Hence, from a theater theoretical perspective “theater in all its manifestations, is essentially concerned with change” (Landy and Montgomery, 2012, p. ix) […] and “when applied to learning, social action and therapy, performance becomes a means for changing understanding, power dynamics, consciousness, and behavior” (Landy and Montgomery, 2012, p. xii). This implies that a theory of change exists in all theater projects regardless of whether that theory is articulated (Kushner et al., 2001), and that all projects are predicated on what someone thinks the participants need and the strategies that will enable them to meet those needs. Importantly, however, theater should not want to change *people*. Instead, as Kushner et al. put it, “theater presents information, emotions, and ideas so to create a condition in which if people wanted to change their ideas or emotional orientations, they could. But the objective is not to change but to invite certain kinds of change” (ibid., p. 66).

There are various examples of how theater has been used for therapeutic purposes to help people to process obstacles in life and strengthen their agency – so-called “therapeutic theater” (Snow et al., 2003; de Aguilera et al., 2018). In one such project, people with psychological problems contributed their narrated experiences to a theater play to be performed by actors. The study participants thus became both constructors and audience, allowing them to gain a deeper understanding of their own psychological condition and to develop coping strategies to adjust to depression, anxiety and distress (Animbom Ngong, 2017).

Theater has also been used with the aim of contributing to interpersonal and social change, not least in work to reduce stigma (Landy and Montgomery, 2012). “Playback theater” was used in a project involving people receiving care within mental health services (Yotis et al., 2017). These individuals were able to stage difficult self-experienced situations to an audience consisting of people who do not usually come into contact with those suffering from psychological ill-health. This theater format, based on the sharing of life experiences of prejudice and discrimination, was discussed as a contributory factor to people taking a stance against existing negative attitudes, and to a decrease in social distancing and the fostering of a community.

In another project, “research-based drama” was used to counteract stigma among people diagnosed with dementia (Mitchell et al., 2019). This performed drama was produced in a collaboration between researchers, theater producers and actors. Like the present study, this performance – which could also be called progressive or political theater – sought to combat stigma by contributing to a better informed understanding among the public of the complexities of the reality of living with dementia. Hence, as the authors put it: “understanding stands in defiance of stigma and can help individuals and societies to see the others’ shared humanity and personhood rather than the difference and judgment” (ibid., p. 22). This research-based drama was shown to have potentially contributed to change on three different levels: first, a changed understanding through the ability to empathize with the other’s perspective; second, an emotional or physical impact, such as feelings of sadness, anger and anxiety, through identification with the protagonists; and, third, changed behavior, demonstrated by reports that audience members would now respond to people with dementia in a different way (ibid.).

## Materials and Methods

### Study Design and Material

Production of the theater play “The abyss: A performance about meeting one’s past” was based on previous research on young people’s narration of their experiences of parental suicide – so-called “research-based theater.” A latent yet clear objective of the play was to inform people about suicide bereavement and to support them to construct destigmatizing understandings of suicide, by highlighting depression and emotional suffering as contributory factors rather than individual responsibility. Hence, the play was assumed to have the potential to assist with psychological processing among the suicide-bereaved and a changed attitude to suicide more generally. Ultimately, the intention was to promote health and wellbeing among those associated with suicide.

The study developed gradually and was carried out in four steps: In a first step, the playwright contacted the author for an interview on her own initiative after having read her previous articles. The playwright’s aim was to inform herself further on the subject as a basis for writing a theater play. In a second step, the playwright wrote the script alone based on what were described as central elements of young people’s parental suicide bereavement experiences. The author of this article had a peripheral role in the production process but was mainly a source of knowledge with no intention other than to provide ideas for inspiration. The idea of the present study, to follow up the play from an audience perspective, arose only after having read the completed script. In a third step, the theater play was organized, staged and rehearsed in collaboration between the playwright, the director and the actors. In the meantime, the author constructed a reflection form with the aim of collecting the audience members’ narrated experiences and thoughts of the theater play as it was performed. In a fourth step, the analysis of the written reflections was conducted – a process that will be described more in detail in the analysis section below.

The play was performed by two female actors at an intimate churchyard theater in Stockholm, Sweden, on 13 occasions during the spring of 2019. The theater environment consisted of a bare stone house surrounded by gravestones. It contained a stripped-down scenography and plastic chairs were lined up for the audience on both sides of the stage floor. The audience consisted of about 40 people and was dominated by women in mid- to late middle age. The play was 1-h long. After the play, the playwright informed the audience about the present study and its aim: to collect and analyze the audience members’ written reflections in order to investigate how they had been affected by a theater performance of this kind. The audience members who wanted to participate in the study were instructed to take an information leaflet and reflection form as they left. The written material encouraged the study participants to reflect freely as all thoughts were welcome, but also to consider *whether* they found the play related to their own lives and, if so, *how*; and *what* they had learned.

Altogether, 41 audience members aged between 37 and 75 (34 women aged 37–75, and 7 men aged 44–67) sent their written reflections to the article author. The reflections differed in length and narrative richness, but it was a rule rather than an exception for there to be a longer written text in which the content of the play was discussed in relation to the person’s own life experience and perspective.

### Ethical Considerations

The study participants’ written reflections are understood as personal material in that they contain descriptions of emotionally sensitive life events and vulnerable situations. At the same time, the participants were informed about the purpose of the study and that the reflections would be published. As a precautionary measure, a decision was taken to alter the ages of the respondents who are quoted, as well as any personal information that might directly reveal their identities.

### A Narrative Analysis of Audience Members’ Storied Responses

The participants’ written reflections of the theater performance were analyzed using narrative methods with the purpose of identifying the central themes and meanings of their content (Riessman, 2008). Accordingly, each reflection was read and analyzed as a narrative whole to see *what* the participants chose to communicate to an imagined reader/the article author based on the questions on the form. Of particular interest in the analysis was to examine *how* suicide was interpreted, given that this study aims to investigate the potential for the integration of alternative destigmatizing meanings of suicide.

The narrative analysis was undertaken in the following three steps. First, the writings were categorized according to the question: “Does the play relate to your own life?”. In response, the participants usually set out their position in relation to suicide in their introduction, as a starting-point for their reflections. Three categories of audience member were thus identified and categorized from their writings: people with their own suicide bereavement experiences, people with experiences of other types of stigmatized trauma and people who did not report any experience of suicide or stigmatized trauma. Second, the written reflections within each category were analyzed more deeply according to the “*How*” aspect of the first question, as well as a second question: “*What* have you learned?”. This analysis revealed what the participants recognized in the play from their own lives, and what they took from it in their current position in relation to suicide or other significant life experience. In the case of the final category, however, the reflections were not primarily related to personal experience. Finally, the participants’ interpretations of suicide were analyzed with regard to the meaning they attributed to it in their reflections; for example, whether suicide was seen as the fault of the suicidal individual or the bereaved family, or caused by other more complex and uncontrollable factors. This was to evaluate whether the participants’ understandings were consistent with a stigmatized attitude to suicide, but also to identify whether any change in attitude was described as having occurred.

### The Storyline: Two Daughters Struggle to Communicate and Resolve the Question of *Why* Their Father Committed Suicide

The theater performance portrayed two sisters – Alice and Theresia – who had never talked about their father’s suicide, which had occurred when they were 13 and 11 years old and led to the creation of a wall of silence in the remaining family. Their father’s suicide was referred to as when he “vanished”. In school and within the family’s social network, the sisters told of how the suicide was surrounded by insecurity and silence.

Alice, the older sister who is portrayed as the responsible and more controlled of the two, discloses to the audience that she has always been jealous of Theresia, who was “daddy’s girl.” While she has only fragmented memories and no photographs of her father, Theresia has several photographs of just her and her father, and she declares that she had felt very close to him. Theresia acts the emotional, care-free and humorous one – perhaps in perceived similarity with their father. The main intrigue revolves around Alice, who is now in her 40s and has begun psychotherapy in an attempt to understand why her father committed suicide. In her search for meaning, she reaches out to Theresia and their mother to start talking about the suicide. However, the performance illustrates the barriers to finding such common ground for communication. Both sisters, in their lonely positions of grief, express deeply embedded feelings of abandonment, yearning, sadness and anger linked to the confusion around how to understand their father’s suicide. The silent interpretation until now had been that their father had *chosen* to commit suicide – that he had made a deliberate decision to leave his daughters and this world behind.

Eventually, the sisters’ searches for meaning are intertwined as they turn to different sources for information. Theresia goes through a bag of the father’s personal belongings and visits his grave to talk to him in a “continued bond” of grief (Klass et al., 1996; Wood et al., 2012). She walks around wearing his shirt and sunglasses in an attempt to reconnect with him and in retrospect to try to read his mind. Alice, on the other hand, reads her father’s hospital records and decides to ask their mother for answers. Her mother’s long-lasting silent meaning-making was that her daughters’ father, who had been discharged from a psychiatric ward on the day of his death, had taken his life because she wanted them to live apart. In a separate scene, the audience gets to know their father before his suicide. He is portrayed as bedridden and severely depressed with no capacity to care for his daughters – Alice almost drowns as a result. The mother is seen as a caring partner who finally reaches the limit of what she can cope with. In the new conversation with Alice, however, the mother paints a different, more amiable, picture of her father – as a funny, smart and wonderful person – when not depressed – who she had loved deeply.

A parallel intrigue emerges as the mother discloses to her daughters that a witness to their father’s suicide had contacted her about giving her a letter in connection with his death, but that she had declined his visit. At the end, the remaining family members in their search for answers decide to meet “the man on the bridge” to hear his story. This now aged man tells how he had tried to convince their father not to jump, but to stay alive. He had clung on to his coat, before their father had struggled free. In the fall, a letter had fluttered out of the coat and landed next to him on the bridge. Now, almost 40 years after the suicide, the three women open the envelope. It contains only an old photograph of the three of them together. On the back is written: “Sorry, I can’t manage anymore. I love you.” The letter allows the daughters to renegotiate their understanding of their father’s suicide: he had no conscious intention to leave them; he loved them but was suffering from severe depression and was desperate to end his emotional pain. In the final scene, the daughters stand together on the bridge. They look down into the abyss and up at the sky and speak out loud, “farewell dad, farewell dad,” in a gesture of reconciliation and parting.

## Results

### The Responses by the Suicide-Bereaved

The analysis of the written reflections by the suicide-bereaved study participants (*N* = 12) showed that their narrative thematic contained two main themes with a strong recognition of their own experience of suicide loss and its effects. These themes were silenced family communication and hindered meaning reconstruction in grief. Their interpretation of suicide was shown in their recapitulation of their lived experience, as well in the interplay with the meanings introduced through the theater performance. Some of their reported thoughts and their own experiences of suicide bereavement are set out below.

#### A Silenced Family Communication Linked to Suicide Being a Stigmatized Death

A major part of these participants’ writings touched on their experience of restricted family communication and the effects it had on them. This 60-year old woman is a case in point. She depicted her lonely position in grief following the traumatic loss of her brother through suicide, and stressed the importance of being able to talk about it.

Thank you, it was a very well-performed play of a trauma that follows you your entire life. Losing a loved one through suicide is so difficult and special. I lost my brother 4 years ago and life has changed a lot. I had always felt like a confident person, but no longer. The worst has happened already, but it is like I always think that something new is going to happen. I am a member of SPES [a Swedish NGO for the suicide-bereaved] and it is very good for me to share what is hard with others who have been affected. There is far too little talk about suicide. When it happened to me, I thought we, my family and friends, would talk about him and how we felt. But that was not the case. Instead, it was expected that you should move on after a while. Sure, life goes on, but it is an unimaginable pain when someone you love is unable to live. What I take with me from the performance is how suicide affects everyone in a whole family, and that you must talk about it and not hide.

In the play, the family’s neighbors gathered in their gardens and chatted over the fence, as they tried to make sense of the suicide by putting all the pieces together in a somewhat sensation-seeking way. This 46-year old woman recognized how she too was affected by such gossip in her social network following her brother’s suicide. In chorus with the previous writer, she emphasized the importance of open family communication, of acknowledging differences in experiences of loss and grief, and of exchanging support, but also of constructing shared memories of the life lived with the deceased.

I think it is very important to highlight siblings’ different experiences of their father’s illness and suicide in this way, but also what the silence can do to you. I recognize so many things, not least the talk among the neighbors and how rumors and stories are created. I think it is very valuable to be able to talk about what happened within the family, and to find a kind of joint strength. Because it is hard to share the experience with someone who has not had the experience and/or who did not know the deceased person. To be able to share the lighter memories is important as well.

This man of the same age lost his father in an accident and later his brother through suicide. He recognized himself in the jealousy conflict between the sisters and reflected on the devastating consequences of unprocessed grief in the family – when it is not possible to reconcile with each other and exchange support.

What I take with me are my own experiences from when my father died (46 years – accident) and how it affected my brother who later committed suicide. The importance of perceived balance and love… A family where jealousy eats you up from inside and makes it difficult to cope with and sort out relations, especially the difficult and complex ones, when they are important…before it is too late.

In the case of this 61-year old woman, her grandmother’s suicide was a well-kept family secret, while her mother’s psychological ill-health was a reality she needed to cope with throughout her childhood. Her reflection contains sadness and disappointment that her mother did not receive the support she needed, through open family communication or professional assistance, to create meaning after her mother’s suicide. This could have prevented a complicated grief from affecting future generations.

I grew up myself with a mother who never got to know whether her mother, my grandma, died by accident or suicide. Mum died some months ago, and her ignorance followed her to the end. The uncertainty and unprocessed grief affected her tremendously and she went through deep depressions, which affected the whole family. With my, even incomplete, knowledge about suicide and psychological trauma, I am convinced that my grandma committed suicide and made it look like an accident. My mum never got to talk to someone professional about her uncertainty and grief. This had devastating consequences, because she was only 14 when she lost her mother. […] A play such as this can facilitate difficult conversations about suicide and help many to understand the underlying causes and thereby support grieving. Thank you!

Similarly, this 72-year old woman described how the theater play opened up closed doors to the suicide secret in her family, which had had a deep impact on her life.

I came into contact with things that have been unmentionable for a long time. My mother’s first husband committed suicide; he shot himself when my oldest sister was a couple of years old. The facade was that he had died of pneumonia. My sister found out the true history by accident. Our aunt mentioned it in passing. Of course, she thought we knew. My sister was then 45 years old. My mother suffered this trauma until her death without telling us children, which of course affected our relationships and our childhoods.

#### A Hindered Meaning Reconstruction in Grief and Theater as Facilitator

Like the sisters in the play, this 55-year old woman described her long struggle to make sense of her father’s suicide in childhood. She underlined her need to recurrently process her paternal loss throughout her life and the healing effect the performance had had on her.

I have my own experience. My dad killed himself when I was 7 years and 10 months old. My 5-years older sister was just 12 years old. I have tried to process the event through therapy and by seeking answers from my mother, while she was alive. It was hard, however, to reach her all the way. I have also written a monolog that was performed in a theater. STILL – this performance resonated with me at various sore points that can still cause pain. The performance did not try to hide anything, play anything or take a stand for right and wrong, which I believe contributed a lot to me being touched – despite having spent YEARS trying to understand what happened in May 1971, and how it has affected my entire life. Having to constantly carry this pain, sore spot in my heart, is and has been very exhausting. It never ends. But it was like a healing, nurturing hand – to see the performance that faced the issue so openly and sincerely. Thank you!

In some cases, it became evident how the play had contributed to an actual *change* in the participant’s understanding of suicide. This 65-year old woman lost her cousin through suicide at a young age. She described how the play made her renegotiate her previous standpoint in relation to suicide; from the interpretation that a dysfunctional and secretive family caused the suicide, to a realization that suicide is caused by depression and unbearable emotional pain. This new destigmatizing interpretation has in previous studies been connected with health-promoting coping strategies, by changing the focus from *who* is to blame for suicide to *what* caused the suicide (Silvén Hagström, 2019). In this case, this new benchmark for reflection on suicide was explained as a potential starting point for reconciliation with the suicide-bereaved family.

It was a strong and well-performed play. I was particularly moved because my cousin took her own life when she turned 20. I really took to heart the idea that you commit suicide because you are sick, under the influence of extreme anxiety and suffering. It was a relief to me. It can dissolve the feelings of guilt and shame that emerge, especially when someone takes their own life at a young age and one wonders if one could have done more to save that person’s life. Perhaps a process of reconciliation has begun with me, especially toward my cousin’s parents. I wanted to find the “guilty” and thought it was a dysfunctional family with secrets. It was perhaps a way to keep the “darkness” away from *me*. Thanks for the play!

This 60-year old mother attended the play with her grown-up daughter on the anniversary of her son’s suicide. He is buried in the cemetery outside the theater. She recognized the experiences of self-blame in grief and said that she perceived the play as very meaningful. There was also an indication that it supported her meaning reconstruction in grief.

So nice (drawn heart) that we could participate. It was the same day 3 years ago that we had the funeral for Lars, Anna’s 3-years younger brother, our forever missed and beloved son. So terribly difficult and sad. […] My son ended up in the abyss. I use the word because it feels true and it is how I have described what happened to him before. Like a steep edge…. He tried following persistent low moods/feelings of depression to commit suicide several times. He died June 21, 2016 and I was the last person who saw him, and also the one who found him and in vain tried to revive him. Darkness…pain. For me it is like his soul has been set free and gained peace. We who remain on earth are the ones who need to struggle. Seeing this play was very meaningful. It is so easy to believe that there is something wrong with you in the wake of such loss. I struggle and learn every day. It is important to take time and not hurry. It was nice for us to come together (drawn heart). Thank you!

Some of the audience members were in their late middle age or older. In some of their reflections, it was emphasized that this kind of insight would have been extra valuable in connection with a parental suicide in childhood or youth, since the majority of their life had passed and been negatively affected by the suicide. This 65-year old woman briefly stated:

I have my own experience of suicide in my family, my father when I was 6 years old. The play felt meaningful and it would have meant a lot to me if I had got so see something similar earlier. A very good play.

In sum, the written reflections by the suicide-bereaved participants show how they felt familiar with the main themes of the play and were supported in grief by the new meaning-makings of suicide that were introduced. These were said to relieve self-accusations and finger pointing in grief. In particular, the suicide-bereaved participants described silenced family communication as the greatest impediment to grieving, since it makes it difficult to share and validate emotions within the family and to construct a common understanding of suicide.

### The Responses by Those Suffering From Other Types of Stigmatized Trauma

Some of the reflections (*N* = 5) were written by people affected by other types of stigmatized trauma, such as having grown up in a family with a parent with a history of mental illness or substance abuse. In these cases, the play was interpreted more broadly as an example of how emotionally challenging and stigmatizing life events can be managed in a family context. In common with the suicide-bereaved participants, the reflections were concentrated on the main theme of a silenced family communication and the subsequent difficulties in comprehending and processing such childhood experiences. In their meaning-making of suicide, these similarities between suicide bereavement and their own traumatic and at the same time stigmatizing experiences are stressed, with reference to blame and shame responses in the family that hinder open communication and prevent access to support.

#### A Silenced Family Communication About Stigmatized Family Troubles

This 54-year old woman recognized the sadness of not being able to have the relationship you had hoped for with your siblings and parents due to stigmatized family troubles. In line with the meaning-making of suicide throughout the play, she underlines the involuntary nature of suicide – that it is never a free choice – and expresses gratitude for the opportunity to reflect and to be introduced to new perspectives.

So much of people’s lives in just 1 h! I recognized myself in most of it. Neither of my parents committed suicide, but they had big problems and issues with mental illness and substance abuse. I recognized myself in the position where you as an adult cannot have the relationships with your siblings and parents that you had hoped for. It feels empty and unsatisfying. As a mourning for the rest of your life where new relationships and attachments are affected. If he chose to take his life, did he also choose his previous depression? The suicide is rather a valve that becomes a permanent solution to a problem that is probably treatable and transient. I believe that the play is about reconciliation …. A warm thank you for this beautiful, high quality and easily accessible performance that gives us the opportunity for important reflections and new perspectives! (drawn flower).

Similarly, this 62-year old woman grew up with a father with an alcohol problem. She recognized the silenced family communication in the play – aiming to conceal the problem – and the sisters’ need to search for answers in relation to their father’s psychological suffering and subsequent death.

I have carried my father’s long-drawn-out suicide with me my whole life. He was an alcoholic. My family couldn’t “save” him. Ola’s [the father’s] behavior in the play reminded me very much about my father’s difficulties talking about his inner life and the humor that existed in parallel to the abyss. Just like Alice, I decided to request my father’s hospital records. Having read them, I got a deeper explanation for and understanding of his behavior. He sought care repeatedly over several years. I was touched to see how he struggled with relapses, denial and confessions, and how he again and again was taken into custody and tried to get out of his alcohol abuse problem. He realized what was happening and said on one occasion after my parents divorced: “Take care of mum, you can’t change me”. He died at 58-years old. I recognize the difficulties in talking about the problem. We fought a lot but there was a lack of dialogue, which is what is most needed in such situations. It became a kind of pact of silence.

From the above, it becomes evident that the grief following a parent’s death linked to long-term alcohol abuse can be likened to a slow suicide by grieving family members, and that there are similarities in the why questions that are raised and have to be addressed as part of the processing of loss. The woman also reported that she had a history of depression and burn out, and that she had been in therapy for several years.

This 45-year old woman experienced comparable family problems in her childhood that could not be talked about. She too emphasized the importance of open and honest family communication where feelings and needs are acknowledged and supported in order to be able to process such adverse childhood events.

I have no personal experience of suicide in my surroundings, but the performance touched me strongly and I cried when the sisters and the mother finally got to say farewell to the father on the bridge. I thought of my own family, how no one spoke about anything and I became sick and felt bad about it; and when the oldest sister in the play told her mother that she was still obsessed with her father’s death, and of her need to express those words and to receive validation. Not arranged, but what really happened. It confirmed me in what I have always had difficulties letting go of/processing in my own family. The importance of confirmation, to have your feelings acknowledged and of being listened to.

The reflections by the participants who had no experience of suicide, but who had suffered from other challenging and stigmatizing family problems, highlighted the similarities between these circumstances. This was particularly the case with restricted family communication – “a pact of silence” – in combination with the need to search for answers, such as the exposed child’s need to process and understand the parent’s psychological problems or substance abuse, which negatively affected the family. In these cases, just like with the suicide-bereaved, the performance was said to be helpful by providing an opportunity to emotionally process past life events and non-stigmatizing explanations for stigmatizing family problems.

### The Responses by Those Not Affected by Suicide or Stigmatized Trauma

A majority of the participants (*N* = 24) reported no experience of suicide or of any other type of stigmatized trauma. Nonetheless, they unanimously underlined the importance of the play, first and foremost in terms of learning from the experiences of others. The narrative thematic consisted of their detailing what they had learned about the complicating aspects of suicide bereavement for the bereaved family. They also paid special attention to how suicide can and should be understood through the meanings introduced in the play as their platform reflection.

#### Learning About the Effects of Suicide on the Bereaved Family

Just like the participants with their own experience of suicide or stigmatized trauma, many of the participants who lacked such experiences learned that suicide can present a barrier to communication. This 57-year old woman told how through the play she had learned about the destructiveness of silence – as an “abyss” – both in relationships and to the *Self*.

The healing and fine ending was the reconciliation between the sisters and their mother. That they could finally meet in grief. The struggle before, between them, was almost more a drama in forgetting and suppressing where they hurt each other and became very lonely, each and every one of them. The culture of silence becomes an abyss itself. Where the sorrow and pain swim like dangerous leviathans under the surface. Powerlessness and self-blame, as a letter in the mail to the family.

This 45-year old woman learned how grief can be managed differently between people – with no rights and wrongs – and how it is never too late to find new meanings in past life events.

A moving story of how a suicide affects a family. It also shows how two siblings, similar in age, who have grown up in the same family can remember so differently. It is portrayed straight, without forcing you to decide who is right. I also take with me the idea of the suicide and the finality of death, and that it is never too late to seek answers to difficult things that have happened.

The power of theater in addressing sensitive life issues, such as suicide bereavement, was discussed by this 72-year old woman. Throughout the performance she was able to identify and empathize with the protagonists and thus process her own loss experiences, but also to become a better informed fellow human being or professional.

I was touched by the genuine tone in the performance. Realizing the importance of theater, music and art that touches the most difficult things in our lives. I have not lost any relatives through suicide, but it is “easy” to identify with because we all experience losses of various kinds […]. Having been in family conversations myself (and still sitting with people in crisis), and even for us professionals working with people who have been through traumatic events, these performances are important. A big THANK YOU for this theater play. So many of us keep our trauma inside – these performances open inner doors, which is the only path to healing. Thanks!

#### Learning About Non-stigmatizing Explanations for Suicide

This 47-year old man learned about the traumatic and stigmatizing aspects of suicide bereavement and reflected on how a culturally marked event – such as suicide – can hinder communication and the reconciliation that can come from shared meaning-making, as with the family portrayed in the play.

This well-written and skillfully staged play led me to think about the traumatic effects of suicide on family members and others. The sisters have internalized these effects and need to articulate and share this experience. The play also raised questions about why people have a tendency to repress or refrain from talking about a suicide (especially if it is culturally marked as shameful, humiliating, immoral). […] Finally, a hopeful ending – the father didn’t just vanish; he was ill, and the daughters could begin to say farewell to him.

Similarly, this 47-year old woman reflected on the meanings of suicide that were introduced by the performance. She learned that suicide is fundamentally an involuntary act: it is not a deliberate choice to die and leave the family behind, it is just not possible to go on living. No one is therefore to blame for suicide.

It was good from the perspective that it is a disease like cancer. It is not that someone wants to leave and die, it is just that they can’t go on living. It is nobody’s fault. You can get angry anyway. You are allowed.

All the participants who did not report any experience of suicide or stigmatized trauma described how through the performance, they had gained new insights into a position as fellow humans and potential support providers, or in a professional role. In line with the above, they learned about the special circumstance of self-blame and stigmatization in suicide bereavement, and several of them repeated the meaning-making that suicide is an involuntary death for which no one is to blame.

### The Role of Theater in Contributing Destigmatizing Meanings of Suicide

In the above, the audience members from all categories showed how they had been made aware through the theater play of the complicating aspects of grief following suicide. In their reflections, the study participants referred to the silenced family communication and the family members’ stigmatized positions in grief as hindering factors in their processing of loss. In their own words, they described how they were emotionally drawn into the plot, and in tandem with the sisters became engaged in their processing of loss and search for meaning as to “why” their father had committed the self-inflicted act of suicide. In this meaning-making process, it became apparent to the participants that all family members carried heavy loads of self-blame, while they themselves through having witnessed the sisters’ struggle and self-questioning approach in grief could not blame them for the suicide. When the family members broke the silence connected to the paternal suicide and opened up to each other, a shared meaning-making became possible in which they could exchange information, liberate each other from guilt and search for shared meaning (Winchester Nadau, 2001; Kaslow et al., 2011). Together, they gathered the strength to face the “man on the bridge” and to open the letter. The study participants’ written reflections revealed that the sisters’ new understanding of their father’s suicide – that he did not choose to leave them behind, that he loved them, but that he had suffered too much to go on living – had been integrated into their understandings of suicide.

The reflections suggest that most participants arrived at the interpretation of suicide as a desperate act and ultimately an involuntary death. Some even demonstrated how the theater play had contributed to a *change* in attitude. That no one with a genuine choice would choose to die seemed to be common ground in this reasoning, which instead focused on different kinds of emotional suffering and depression as contributing circumstances to suicide. In addition, in a dialog between Alice and the mother in the play the father’s mental health problems were externalized from who he was as person. The father was depicted as deeply affected by depression during his final months, but the mother declared that this was not who their father was. Instead, he was depicted as a “funny, smart and wonderful person”. In this way, the theater play not only liberated the suicidal individual and his family from responsibility for suicide, but also acknowledged the memory construction of *who* the deceased parent was separate from suicide as an important part of this liberation and the daughters’ grieving process (Nickman et al., 1998). One 45-year old woman, who had not herself been affected by suicide, summed up her central understandings from the play. These are representative of most of the participants and highlight the role of and potential for theater to contribute destigmatizing meanings to counteract culturally stigmatizing notions of suicide.

[I am] grateful for this fine-tuned and emotional play about how difficult it can be to cope with grief when a loved one commits suicide. The taboo aspect, common prejudices that the neighbors with their lawnmowers represent…. Not daring to talk about it, not being able to mourn. The disease aspect, i.e. often depression…. It is also difficult for psychiatry to identify. It warms the frozen landscape to also remember the deceased by suicide for his bright, fine sides.

This shows how the narrative points from the theater performance have the potential to affect culturally charged notions of suicide and help to counteract the stigmatizing attitudes that burden and complicate the grieving of suicide-bereaved family members. Many of the study participants reported that they could identify with the position of the protagonist sisters; either by being suicide-bereaved themselves or by finding themselves in a position defined by stigmatization and silence due to other family troubles. However, those who did not report any experience of suicide or other types of stigmatized trauma also seem to have empathized with the sisters as they expressed how they had been emotionally moved by the performance. This is in line with Mitchell et al. (2019) use of research-based drama. Just as in their study, the audience was introduced to alternative understandings of a stigmatized subject based on research, through an emotionally charged experience and identification with the protagonist. This theater format made a potential change in audience behavior possible. Such integrated learning through theater – between cognitive meaning reconstruction and an emotionally based experience – therefore seems to constitute a particularly favorable condition for the internalization of new understandings.

## Discussion

### Discussion of Main Results

This article has sought to investigate how a theater play might counteract the stigmatization of suicide bereavement and contribute new understandings of suicide. The theater format analyzed was inspired by previous research about young people’s experiences of grieving after a parent’s suicide – so-called research-based theater. Special attention in the play was paid to the two narrator sisters’ different grief responses, their concurrent preoccupation with *why* their father had committed the self-inflicted act of suicide, and their persistent struggle to search for answers. Hence, it proved difficult to construct a manageable meaning from their stigmatized family situation, where the suicide had been encapsulated in silence.

The audience members’ written reflections after witnessing the play were free in nature, but at the same time structured based on the questions of *whether* the play related to their own lives, and if so *how*; and *what* they had learned. The results showed that many of the suicide-bereaved participants empathized with the mourning daughters and recognized themselves in their lonely position in grief, as well as their struggle to search for answers in relation to the suicide stigma. In their written reflections, the negative consequences of silenced or conflicted family communications after suicide were detailed from their own experiences. It was underlined how open and honest family communication – where feelings and needs can be shared and validated, and the memories and meanings of the deceased family member’s life and death jointly constructed – was attributed the greatest importance to the ability to process the suicide loss (c.f. Winchester Nadau, 2001; Kaslow et al., 2011). Such family communication stands in sharp contrast to what seemed to be the dominant experience among the suicide-bereaved participants. This was something that many expressed a hope for and would probably also have benefitted from in their grief.

The experience of thwarted family communication was shared by the participants who reported experiences of other forms of stigmatized trauma, such as mental illness and substance abuse in a parent. This finding is consistent with research on the stigma connected to both mental illness and substance abuse (Kroll, 2004; Larson and Corrigan, 2008; Moses, 2014; Silvén Hagström and Forinder, 2019). Those participants also expressed a strong desire to talk about their disadvantaged childhoods, to renegotiate meaning and to restore moral identities in the wake of stigmatized trauma. In both cases, experiences of abandonment, sadness, anger and confusion dominated the childhood narratives, with the risk of long-term effects from self-blame and shame responses. The third category of participants differed from the previous two, since they did not report any experience of either suicide or other types of stigmatized trauma. However, the performance was discussed as a means of processing other types of more “normal” loss experiences in a shared community of grieving. More importantly, given the focus of this study, they also reported that they had learned something new about the distinguishing characteristics of suicide bereavement. It was said that this new knowledge would contribute to a more informed position in their future contacts with people bereaved through suicide.

Finally, participants from all categories reported how they had learned that suicide is a desperate rather than a deliberated act, mainly linked to overwhelming emotional pain or depression. In the end, suicide was thus perceived as an involuntary act caused by complex and interacting factors linked to both inner vulnerabilities and stressful life events, and an act for which no one was to blame (c.f. Silvén Hagström and Forinder, 2019). This destigmatizing interpretation of suicide is in line with the sisters’ narrative meaning reconstruction in the performed theater play. Consequently, this demonstrates how these understandings have been integrated and made conscious by the audience. Sudak et al. (2008) suggests that it is beneficial for the suicide-bereaved to receive non-stigmatizing explanations for suicide in a professional contact, such as that depression is a contributory factor, to relieve self-blame in relation to the “why-question” in grief. They also recommend that such explanations are provided to educate the public in order to decrease stigma, both through a change in general attitudes and to counteract internalized stigma among the suicide-bereaved. This study represents one such educational effort to inform the public and individual mourners about suicide bereavement and non-stigmatizing explanations for suicide.

### Limitations

The present study is limited in that a large proportion of the audience chose not to participate by providing their reflections. This could indicate that the results are biased in that they are only based on the reflections of the participants who were motivated enough to contribute their views on the play and their personal life experience to the research. In the case of the suicide-bereaved participants, and those affected by other types of stigmatized trauma, this would be in close conformity with the results of wider research. Hence, respondents expressed a strong desire to talk about previously silenced life experiences, and the theater performance was seen as assisting with doing just that. From this reasoning, it can be assumed that the audience members who declined to participate in the study chose to do this on the basis that the performance either touched on experiences that they still found too difficult to articulate, or that they simply wanted to see a theater play and not to participate in a research study.

## Conclusion

Regardless of the limitations noted above, the study results contribute to the research field of suicide postvention and prevention: first, by validating previous research findings on a culturally induced suicide stigma (Cvinar, 2005; Feigelman et al., 2009; Jordan and McIntosh, 2011; Silvén Hagström, 2019); and, second, by illustrating and analyzing how research-based theater could function as a means for combating this stigma. The results point to the conclusion that research-based theater is a time-limited and cost-effective method of introducing alternative meanings and identities to both individual mourners and the broader cultural context from which stigma originates. They also show how it can have destigmatizing effects on a stigmatized trauma such as suicide bereavement. A recommendation for future research, as well as practice that includes the vulnerable group of suicide-bereaved family members or other stigmatized groups, would be to investigate further how research-based theater could be used to counter stigmatization and contribute to psychological processing of traumatic life events in other contexts. Many other initiatives and strategies are also needed to combat suicide stigma as part of a health-promotion approach to suicide bereavement. For example, there are several examples of how other narrative methods can support people individually or in groups to reconstruct meanings and identities in relation to a suicide loss in the family (see for example Sands, 2009; Stepakoff, 2009; Thompson and Neimeyer, 2014; Sather, 2015; Silvén Hagström, 2017). All such efforts to assist in the construction of destigmatized meanings and moral identities in the wake of suicide have the potential to help the suicide-bereaved to experience post-traumatic growth, and thus improved health and wellbeing (Neimeyer, 2004).

## Data Availability Statement

The datasets generated for this study are available on request to the corresponding author.

## Ethics Statement

Ethical review and approval was not required for the study on human participants in accordance with the local legislation and institutional requirements. The patients/participants provided their written informed consent to participate in this study. Written, informed consent was obtained from the individual(s) and/or minor(s)’ legal guardian/next of kin for the publication of any potentially identifiable images or data included in this manuscript.

## Author Contributions

The author is the sole researcher in the project and responsible for the study process from material gathering to analysis and manuscript writing.

## Conflict of Interest

The authors declare that the research was conducted in the absence of any commercial or financial relationships that could be construed as a potential conflict of interest.
